# Complications after surgical treatment of acetabular fractures: a 5-year follow-up of 229 patients

**DOI:** 10.1007/s00590-022-03284-1

**Published:** 2022-05-20

**Authors:** Natalie Lundin, Hans E. Berg, Anders Enocson

**Affiliations:** 1 Department of Trauma, Acute Surgery and Orthopaedics, Karolinska University Hospital, 171 64 Stockholm, Sweden; 2 Department of Molecular Medicine and Surgery, Karolinska Institute, Stockholm, Sweden; 3 Division of Orthopedics and Biotechnology, Department of Clinical Science, Intervention and Technology, Karolinska Institute, Stockholm, Sweden

**Keywords:** Acetabular fracture, Trauma, Epidemiology, Surgical treatment

## Abstract

**Purpose:**

Acetabular fractures are injuries often surgically treated, but the surgical intervention is associated with a high risk of subsequent complications. The primary aim of this study was to explore the rate of reoperations and to identify potential risk factors for reoperation. Secondary aims were other adverse events and mortality.

**Methods:**

Patients ≥ 18 years with a surgically treated acetabular fracture at a single trauma center in Sweden between 2010 and 2019 were retrospectively included. Data were collected through review of medical records and radiographs. Logistic regression analysis was performed to investigate factors associated with reoperations and other adverse events.

**Results:**

A total of 229 patients with a surgically treated acetabular fracture were included, mean age (± SD, range) 60 (19, 19–94) years. The majority of the patients were males (*n* = 180, 79%), and the median (IQR) follow-up time was 1779 (1906) days (4.9 years). 47 patients (21%) underwent a reoperation. THA as surgical method was associated with a lower reoperation rate compared to ORIF (OR 0.3, 95% CI 0.1–0.8, *p* < 0.01). 72 patients (31%) sustained an adverse event not requiring reoperation, and admittance to ICU was associated with an increased risk (OR 2.6, 95% CI 1.2–5.7, *p* = 0.02). 30-day mortality was 3.1% and 1-year mortality 5.7%.

**Conclusion:**

The complication rate after acetabular fracture surgery was high, and surgical treatment with primary THA was associated with a reduced risk for reoperation.

## Background

Acetabular fractures are complex articular injuries mainly encountered among older patients after simple falls, but younger individuals may also be affected after high-energy trauma [1]. Although sometimes labeled a pelvic fracture, the acetabular fracture should preferably be described as a separate entity due to its articular engagement, and the specific characteristics regarding epidemiology and treatment. The acetabular fracture is less common than the pelvic fracture with incidence rates between 5 and 11/100,000 person-years in recent reports, and males are more frequently affected than females across all ages [1–4]. Surgical treatment is more common for acetabular than for pelvic fractures, and 14–15% of all patients are treated surgically [3, 4]. Current literature proposes increasing incidence rates of acetabular fractures, mainly in the older population, suggesting a potential upsurge of patients requiring acetabular surgery [1, 3, 4].

Surgical treatment of acetabular fractures aims at restoring joint congruency and if possible, preserve the native hip joint. This can be achieved by open reduction and internal fixation in patients with adequate bone quality. Another option is primary joint replacement, with or without simultaneous fracture stabilization, and this option might be more suitable for older patients with complex fracture patterns or insufficient bone quality [5, 6]. The complication rate after surgical treatment is known to be high with reported reoperation rates of 15% [7] and overall complication rates of 21% [8]. However, few studies with larger number of patients exist, and the available literature mainly investigates patients treated with either osteosynthesis or joint replacement separately.

The aim of this study was to explore the complication rate after surgical treatment of acetabular fractures in an unselected cohort of patients. Our primary outcome was reoperation rate, and secondary outcomes were other adverse events and mortality.

## Methods

### Study population

In this retrospective study, we included all patients ≥ 18 years surgically treated for an acetabular fracture at the Karolinska University Hospital in Stockholm, Sweden, between 2010–2019. Patients were identified through the local surgical planning software, and variables were collected by reviewing medical charts, including radiographs. Patients with combined pelvic- and acetabular fracture were not included. Follow-up time was from injury date until December 31, 2020, or death. Permission from the National Ethics Agency was granted with reference number Dnr 2019–05846.

### Variables

Collected demographic variables were age, gender, and American Society of Anesthesiologists (ASA) classification. Injury variables were injury mechanism, vital parameters upon arrival (systolic blood pressure and heart rate), Glasgow Coma Scale (GCS), hemoglobin level at arrival, concomitant injuries, and fracture type. Fractures were classified according to the Judet and Letournel classification [9] which comprises ten fracture patterns: five simple and five complex fracture types. Fractures were classified by reviewing the classification performed by the operating surgeon preoperatively and confirmed by the reviewing of radiographs by two of the authors together (NL and AE). Treatment variables were time to surgery, method of surgical treatment, hospital length of stay and intensive care. Follow-up variables were unplanned reoperations, other adverse events not requiring reoperation (nerve injury, pneumonia, pulmonary embolism, urinary tract infection, deep venous thrombosis) and mortality.

### Statistics

Numerical data were presented as mean (± SD, range) or median (IQR or IQR, range). Categorical data were presented as frequency and percent distribution. Logistic regression analysis was done to investigate potential factors associated with unplanned reoperation or other adverse events. At first, crude association for each variable was tested in univariable models. Subsequently, a multivariable model was used to study the adjusted associations. The associations were presented as odds ratios (ORs) with 95% confidence intervals (CIs). The results were considered significant at *p* < 0.05. The statistical software used was IBM SPSS Statistics, Version 25 for Windows (SPSS Inc., Chicago, Illinois).

## Results

### Epidemiology

A total of 229 adult patients were surgically treated for an acetabular fracture during the 10-year long study period (2010–2019). The mean age (± SD, range) was 60 (19, 19–94) years and the majority of the patients were males (*n* = 180, 79%). Median (IQR) follow-up time was 1779 (1906) days (4.9 years). The most common injury mechanism was a simple fall in the same level (*n* = 83, 36%), followed by a car accident (*n* = 42, 18%) and a fall from height (14%, *n* = 31). 57% of the patients (*n* = 130) had sustained their acetabular fracture through a high-energy mechanism, and 2.6% (*n* = 6) were reported to have an intentional cause (suicide attempt) of injury (Table 1).

**Table 1:** Epidemiology, vital parameters on arrival, time to surgery and length of stay

Variable	All patients n = 229
Age; Mean (± SD, range)	60 (19, 19–94)
Age ≥ 60; *n* = (%)	124 (54)
Gender female; *n* = (%)	49 (21)
ASA-class 3–4; *n* (%)	79 (35)
*Injury mechanism; n* = *(%)*	
Simple fall	83 (36)
High fall	31 (14)
Car related	42 (18)
Motorcycle related	14 (6.1)
Other traffic related	30 (13)
Other	29 (13)
High-energy trauma mechanism; *n* = (%)	130 (57)
Intentional cause of injury; *n* = (%)	6 (2.6)
GCS; median (IQR)	15 (0)
GCS < 9; *n* = (%)	7 (3.1)
SBP (mmHg); median (IQR)	130 (25)
Shock; *n* = (%)	1 (0.5)
Pulse rate; median (IQR)	80 (20)
Hb (g/L); Median (IQR)	124 (24)
Head or neck injury; *n* = (%)	24 (11)
Chest injury; *n* = (%)	45 (20)
Abdominal injury; *n* = (%)	9 (3.9)
Major spine injury; *n* = (%)	14 (6.0)
Major upper limb injury; *n* = (%)	24 (11)
Major lower limb injury; *n* = (%)	26 (11)
Dislocated hip; *n* = (%)	55 (24)
Time to surgery (days); median (IQR)	3.0 (3)
Hospital length of stay (days); median (IQR)	8.0 (7)
ICU care; *n* = (%)	33 (14)
ICU care length of stay (days); median (IQR)	5.0 (11)

*IQR* interquartile range, *ASA* American Society of Anesthesiologists, *GCS* Glasgow Coma Scale, *SBP* systolic blood pressure, *Shock* SBP < 90 mmHg, *Hb* hemoglobin, *ICU* intensive care unit

Almost all patients presented with stable vital parameters upon arrival to the hospital (Table 1.) However, 67% (*n* = 153) of the patients were referred from other hospitals and might thus have had deviating vital parameters primarily on arrival at the local hospital. Concomitant injuries occurred with the most common being a chest injury (*n* = 45, 20%). Almost one in four patients (*n* = 55, 24%) had a dislocated hip at the time of the first x-ray (Table 1).

Median (IQR) time to surgery was 3 (3) days and median (IQR) hospital length of stay was 8 (7) days. 14% of the patients (*n* = 33) needed intensive care (Table 1).

### Fracture classification and treatment

The most common acetabular fracture types in this study, classified according to Judet and Letournel, were anterior column + posterior hemi transverse (22%, *n* = 50), associated both column (21%, *n* = 49) and posterior wall (20%, *n* = 45). One patient with solitary fracture of the quadrilateral plate did not fit into any of the ten fracture types and was counted as *unable to classify*. The main treatment method was open reduction and internal fixation (ORIF) with plating and screws only (74%, *n* = 169). A total of 54 (24%) patients were treated with a total hip arthroplasty (THA); THA + cage (*n* = 30, 13%), THA + cage + plating (*n* = 21, 9.2%) and THA only (*n* = 3, 1.3%) (Table 2).

**Table 2:** Type of acetabular fracture in relation to treatment

	Type of treatment; *n* = (%)					Incision, *n* = (%)		
Fracture type; *n* =	ORIF *n* = (%)	THA *n* = (%)	THA + cage *n* = (%)	THA + cage + plating *n* = (%)	Other *n* = (%)	Anterior approach	Posterior approach	Combined approach
Anterior column + posterior hemi transverse; 50 (22)	29 (58)	1^a^ (2.0)	13 (26)	7 (14)	0	38 (76)	8 (16)	4 (8)
Associated both column; 49 (21)	35 (71)	0	4 (8.2)	10 (20)	0	21 (43)	12 (24)	16 (33)
Posterior wall; 45 (20)	40 (89)	1^a^ (2.2)	0	0	4 (8.9)	1 (2.2)	44 (98)	0
Anterior column; 35 (15)	24 (69)	1 (2.9)	8 (23)	1 (2.9)	1 (2.9)	32 (91)	2 (5.7)	1 (2.9)
Transverse + posterior wall; 18 (7.9)	17 (94)	0	0	1 (5.6)	0	3 (17)	12 (67)	3 (17)
Anterior wall; 9 (3.9)	5 (56)	0	3 (33)	0	1 (11)	8 (89)	1 (11)	0
Posterior wall + posterior column; 9 (3.9)	8 (89)	0	1 (11)	0	0	0	9 (100)	0
T-shaped; 6 (2.6)	4 (67)	0	0	2 (33)	0	1 (17)	4 (67)	1 (17)
Transverse; 5 (2.2)	4 (80)	0	1 (20)	0	0	2 (40)	3 (60)	0
Posterior column; 2 (0.9)	2 (100)	0	0	0	0	0	2 (100)	0
Unable to classify; 1 (0.4)	1 (100)	0	0	0	0	1 (100)	0	0
All; 229 (100)	169 (74)	3 (1.3)	30 (13)	21 (9.2)	6 (2.6)	107 (47)	97 (42)	25 (11)

*ORIF* Open reduction internal fixation, *THA* total hip arthroplasty

^a^Treated with THA + additional plating

Nine patients had a Pipkin fracture type IV [10]. Four of these patients with concomitant posterior wall fracture had fracture fragments fixated with screws only, bone anchors or only fracture fragment removal without fixation, and they were listed as treated with “other” method in Table 2. One patient with an anterior column fracture was treated with screws only, and was also listed as “other” treatment, as was one patient with anterior wall fracture who suffered cardiac arrest at the operating table before internal fixation was performed (Table 2).

### Reoperations

**Fig. 1:**
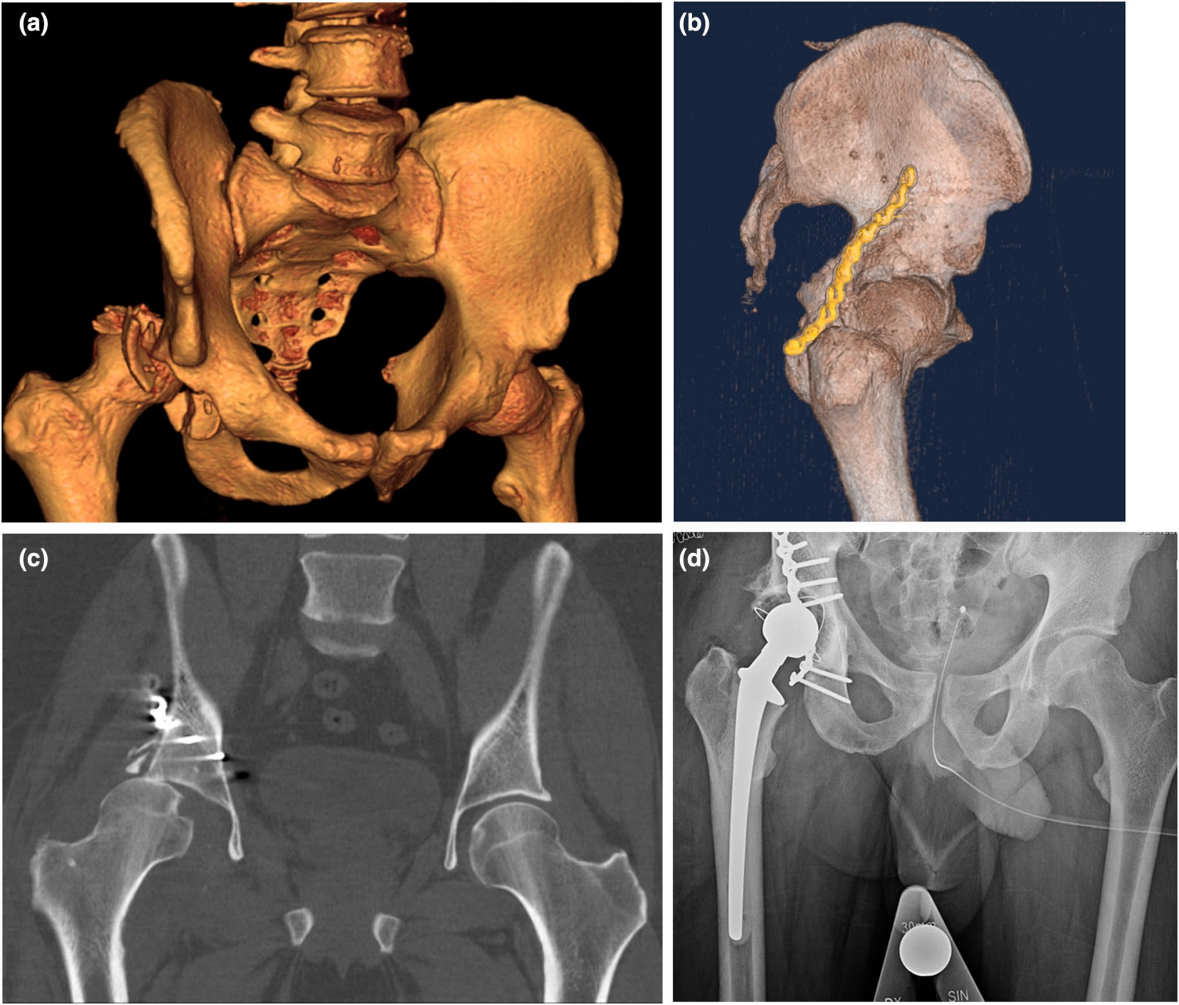
**a** Computed tomography (CT) scan of a 45-year-old male who sustained a posterior wall acetabular fracture with dislocated hip after a high energy car accident. **b** Postoperative CT scan after treatment with plate fixation. **c** CT scan after 6 weeks showing osteosynthesis failure with subluxation of the femoral head. **d** Postoperative radiographs after reoperation with a cemented THA

47 patients (21%) underwent a reoperation after the primary surgery. The main indication for reoperation was arthrosis (*n* = 17, 7.4%), followed by infection (*n* = 9, 3.9%) (Fig. 1a–d, 2a–d) (Table 3). All patients reoperated due to arthrosis received a secondary THA, as did two additional patients during their second reoperation (first reoperation due to osteosynthesis failure). None of the patients reoperated due to infection had sustained a PJI (Prosthesis joint infection). Six patients (2.6% of the entire cohort and 11% of the patients treated with primary THA) had a dislocation of the THA, of which one underwent revision surgery with change of implant. All six patients reoperated due to dislocated hip were initially operated with a posterior hip approach. 12 patients (5.2%) were reoperated due to indications possible to classify as “avoidable” or due to “surgeons’ error,” and these included malplaced implant, osteosynthesis failure and persisting loose bone fragments in the joint. Time to the first reoperation varied considerably with the different indications (Table 3). 12 patients (5.2%) underwent more than one reoperation, and the main indications for repeated reoperations were infection, dislocated THA or arthrosis. Logistic regression analysis was performed to evaluate potential factors associated with risk for reoperation. Age (≤ 60 or > 60 years (median age), gender (female or male), acetabular fracture hip dislocation (yes or no, as a proxy for a more severe injury), admittance to intensive care unit (yes or no) and surgical method (ORIF or THA) were tested. Male gender was associated with a reduced risk for reoperation compared to females in the multivariable analysis (OR 0.4, 95% CI 0.2–0.9, *p* = 0.02). THA as surgical method was associated with a lower reoperation rate compared to ORIF in the multivariable analysis (OR 0.3, 95% CI 0.1–0.8, *p* = 0.01) (Table 4).

**Fig. 2:**
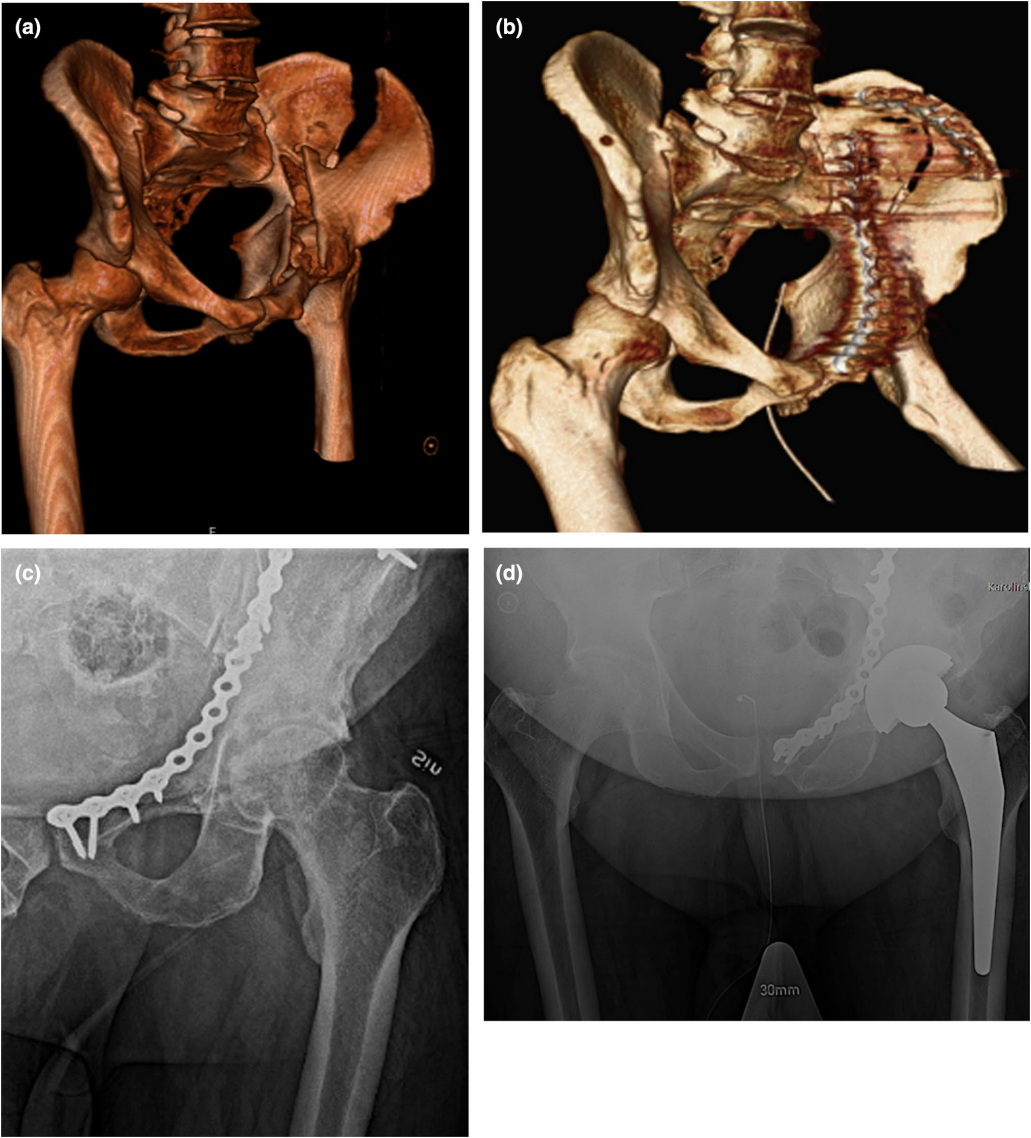
**a** CT scan of a 62-year-old male who sustained an anterior column + posterior hemi transverse acetabular fracture after a low-energy fall. **b** Postoperative CT scan after treatment with plate fixation. **c** Radiograph 3 years later displaying signs of arthrosis and avascular necrosis of the femoral head. **d** Postoperative radiographs after reoperation with an uncemented THA

**Table 3:** Indication for first reoperation

Indication	Patients *n* = (%)	Time (days) to reoperation Median (range)
Arthrosis	17 (7.4)	342 (114–1103)
Infection	9 (3.9)	23 (11–80)
PJI	0 (0)	0 (0)
Dislocated THA	6 (2.6)	13 (1–314)
Malplaced implant	5 (2.2)	6.0 (3–52)
Osteosynthesis failure	4 (1.7)	36 (16–97)
Loose bone fragments in joint	3 (1.3)	4 (3–5)
Disturbing implant	3 (1.3)	352 (322–564)
All	47 (21)	80 (1–1103)

*PJI* Prosthesis joint infection

**Table 4:** Logistic regression to evaluate factors associated with risk for reoperation

Variable	All *n* =	Reoperation	Univariable	Multivariable		
		*n* = (%)	OR (95%CI)	*p*-value	OR (95%CI)	*p*-value
*Age*						
≤ 60 years	105	20 (19)	1 (reference)		1 (reference)	
> 60 years	124	27 (22)	1.2 (0.6–2.3)	0.6	1.6 (0.8–3.5)	0.2
*Gender*						
Female	49	15 (31)	1 (reference)		1 (reference)	
Male	180	32 (18)	0.5 (0.2–1.0)	0.05	0.4 (0.2–0.9)	**0.02**
*Hip dislocation*						
No	174	34 (20)	1 (reference)		1 (reference)	
Yes	55	13 (24)	1.3 (0.6–2.6)	0.5	1.1 (0.5–2.5)	0.8
*ICU*						
No	196	38 (19)	1 (reference)		1 (reference)	
Yes	33	9 (27)	1.6 (0.7–3.6)	0.3	1.6 (0.7–3.9)	0.3
*Surgical method*						
ORIF	175	41 (23)	1 (reference)		1 (reference)	
THA	54	6 (11)	0.4 (0.2–1.0)	0.06	0.3 (0.1–0.8)	**0.01**

*OR* odds ratio, *CI* confidence interval, *ICU* intensive care unit, *ORIF* open reduction internal fixation, *THA* total hip arthroplasty. Results were considered significant at *p* < 0.05

### Other adverse events and mortality

31% (*n* = 72) of the patients suffered an adverse event not requiring reoperation. The most common adverse event was nerve injury (*n* = 27, 12%), followed by pneumonia and pulmonary embolism at 8.3% (*n* = 19) and 5.7% (*n* = 13), respectively (Table 5). Approximately half of the patients with postoperative nerve injury (*n* = 14) had merely sustained loss of function of the lateral femoral cutaneous nerve, a sensory nerve deficit often associated with the skin incision. All other nerve injuries (*n* = 13) constituted loss of function related to the sciatic nerve. 30-day mortality was 3.1% (*n* = 7) and 1-year mortality 5.7% (*n* = 13). Logistic regression analysis was performed to evaluate potential factors associated with risk for these other adverse events. Age (≤ 60 or > 60 years (median age), gender (female or male), acetabular fracture hip dislocation (yes or no), admittance to intensive care unit (yes or no) and surgical method (ORIF vs THA) were tested. Admittance to ICU was associated with an increased risk for other adverse events in both the univariable (OR 2.5, 95% CI 1.1–5.0, *p* = 0.03) and multivariable (OR 2.6, 95% CI 1.2–5.7, *p* = 0.02) analysis (Table 6).

**Table 5:** Adverse events not requiring reoperation

Adverse event	Patients *n* = (%)
Nerve injury	27 (12)
Pneumonia	19 (8.3)
PE	13 (5.7)
UTI	10 (4.4)
DVT	6 (2.6)
Superficial wound infection	5 (2.2)
Kidney failure	2 (0.9)
Sepsis	2 (0.9)
All	72 (31)

*PE *pulmonary embolism, *DVT* deep venous thrombosis, *UTI* urinary tract infection

**Table 6:** Logistic regression to evaluate factors associated with adverse event not requiring reoperation

Variable	All *n* =	Reoperation	Univariable	Multivariable		
		*n* = (%)	OR (95%CI)	*p*-value	OR (95%CI)	*p*-value
*Age*						
≤ 60 years	105	32 (31)	1 (reference)		1 (reference)	
> 60 years	124	40 (32)	1.1 (0.6–1.9)	0.8	1.1 (0.5–2.1)	0.9
*Gender*						
Female	49	14 (29)	1 (reference)		1 (reference)	
Male	180	58 (32)	1.2 (0.6–2.4)	0.6	1.1 (0.6–2.4)	0.7
*Hip dislocation*						
No	174	57 (33)	1 (reference)		1 (reference)	
Yes	55	15 (27)	0.8 (0.4–1.5)	0.4	0.7 (0.3–1.4)	0.3
*ICU*						
No	196	56 (29)	1 (reference)		1 (reference)	
Yes	33	16 (49)	2.5 (1.1–5.0)	**0.03**	2.6 (1.2–5.7)	**0.02**
*Surgical method*						
ORIF	175	54 (31)	1 (reference)		1 (reference)	
THA	54	18 (33)	1.1 (0.6–2.1)	0.7	1.1 (0.5–2.4)	0.8

*OR *odds ratio, *CI *confidence interval, *ICU *intensive care unit, *ORIF *open reduction internal fixation, *THA *total hip arthroplasty. Results were considered significant at *p* < 0.05

## Discussion

Our main finding was a high reoperation rate (21%) in patients surgically treated for an acetabular fracture. Female gender was found to be a risk factor for reoperation (31% vs. 18%), and patients treated with primary THA had a lower reoperation rate (11%) than patients treated with ORIF (23%).

Reoperation rate after acetabular surgery is known to be high with reported rates of between 8 and 19%, and hence, our findings are slightly higher than previously reported [7, 11–14]. However, most existing studies investigate patients treated with open reduction and internal fixation only and not a combined cohort of patients treated primarily with either ORIF or THA as in our study. This is an important aspect since the primary indication for reoperation in existing literature as well as in our study was the development of secondary arthrosis and conversion to THA. An American study followed a large number of surgically treated acetabular fracture patients and found a reoperation rate of 15%, lower than in our material. [7]. They did, however, investigate a younger cohort of patients, after only ORIF, with a mean age of around 40 years (compared to 60 years in our study), and age has previously been suggested as a negative predictor for joint survival after acetabular fracture surgery [13], although we did not find any association between older age and an increased rate of reoperation in this study. Another discrepancy between our cohort and the patients in the study by Ding et al. was the difference in fracture types; the most common acetabular fracture type in our material was the anterior column + posterior hemi transverse, a fracture pattern commonly seen among elderly with osteopenia [15], compared to the posterior wall fracture which was the most common in the study by Ding et al. The main reason for the higher reoperation rate found in our study can also be explained by the longer follow-up time in our study (median 4.9 years) compared to an average of 52 weeks in the study by Ding et al., and some patients might not have developed arthrosis requiring surgery yet after that time.

Earlier studies suggest that increasing age, hip dislocation and certain fracture types might be associated with an increased risk for reoperation [7, 13]. Female gender has to our knowledge not previously been described as a risk factor for reoperation. This finding must, however, be valued with some precaution since only 21% of the patients in our material were females, a somewhat lower number compared to other cohorts of surgically treated acetabular fracture patients, where the proportion of females often is slightly higher at rates around 30% [7, 12]. One hypothesis regarding a potential increased risk for reoperation among females might be the anatomical variances and potential bone quality difference in the male compared to the female pelvis, which might complicate the surgery of females with acetabular fractures. This issue demands analysis and should be studied further.

The most common indication for reoperation in the literature was as previously mentioned arthrosis with subsequent conversion to THA, which affects 5.7–15% of the patients [7, 12–14]. Our conversion rate of 7.9% spans in the lower limit compared to previous data, although of course 24% of the patients in our material were already treated with a primary THA. It is known that primary THA as treatment for acetabular fracture has a good outcome regarding implant survival and the need for revision surgery has been reported to be low, especially compared to later surgical treatment with THA after failed ORIF [5, 16, 17, 19]. Our results support this knowledge where patients treated with a primary THA had a low reoperation rate. In our material, only one patient required revision surgery after primary THA, with exchange of implant due to dislocated hip prosthesis. These findings suggest that there might be reason to consider primary THA to a greater extent for certain acetabular fracture patients.

Reoperation with debridement due to deep infection was not common in our study (3.9%) and in the lower range of what has previously been reported (3.9–7%) [7, 18].

Common other adverse events for patients with surgically treated acetabular fractures are iatrogenic nerve injuries, thromboembolic events, bleeding, and pneumonia [8, 12]. We found 12% of the patients sustaining a nerve injury, whereof approximately half of them only experienced a sensory loss related to injury of the lateral femoral cutaneous nerve. In the meta-analysis by Giannoudis et al. covering over 3000 acetabular fractures, nerve injury affected 8–16% of patients surgically treated for acetabular fracture. This highlights a rather substantial injury risk important to communicate to a patient awaiting acetabular surgery. Another well-known common adverse event is the risk of thromboembolic events which has been reported to affect around 4–5% [8, 12]. We found a slightly higher rate (8%) in our material, and this could possibly be explained by our somewhat older patient-cohort. There was an association between ICU admittance and an increased risk for adverse events not requiring reoperation, which is previously described and probably reflects a more severely injured or fragile patient [7].

Mortality after surgical treatment of acetabular fracture is hardly reported previously, and our rates of 3.1% at 30 days and 5.7% at 1 year are considered relatively low considering our patient cohort.

### Strengths and limitations

The major strength of this study was the unselected study population with a large cohort of surgically treated acetabular fracture patients. The comparably long follow-up time of almost five years displays a hopefully proper rate of both short- and long-term complications compared to previous studies. A main limitation was of course the retrospective design where we cannot rule out that certain complications could have been overlooked if the patient for example received medical care for a complication at another caregiver than at the hospital of treatment. We also lack any clinical follow-up with functional status, etc.

## Data Availability

Not applicable.

## References

[1] Rinne PP, Laitinen MK, Huttunen T, Kannus P, Mattila VM (2017). The incidence and trauma mechanisms of acetabular fractures: a nationwide study in Finland between 1997 and 2014. Injury.

[2] Best MJ, Buller LT, Quinnan SM (2018). Analysis of incidence and outcome predictors for patients admitted to US hospitals with acetabular fractures from 1990 to 2010. Am J Orthop (Belle Mead NJ).

[3] Melhem E, Riouallon G, Habboubi K, Gabbas M, Jouffroy P (2020). Epidemiology of pelvic and acetabular fractures in France. Orthop Traumatol Surg Res.

[4] Lundin N, Huttunen TT, Berg HE, Marcano A, Felländer-Tsai L, Enocson A (2021). Increasing incidence of pelvic and acetabular fractures. A nationwide study of 87,308 fractures over a 16-year period in Sweden. Injury.

[5] Enocson A, Blomfeldt R (2014). Acetabular fractures in the elderly treated with a primary Burch–Schneider reinforcement ring, autologous bone graft, and a total hip arthroplasty: a prospective study with a 4-year follow-up. J Orthop Trauma.

[6] Butterwick D, Papp S, Gofton W, Liew A, Beaulé PE (2015). Acetabular fractures in the elderly: evaluation and management. J Bone Joint Surg Am.

[7] Ding A, OʼToole RV, Castillo R, Reahl B, Montalvo R, Nascone JW, Sciadini MF, Carlini AR, Manson TT (2018). Risk factors for early reoperation after operative treatment of acetabular fractures. J Orthop Trauma.

[8] Küper MA, Trulson A, Stuby FM, Stöckle U, Konrads C (2020). Complications of surgical approaches for stabilization of pelvic ring injuries: Analysis of pitfalls and how to avoid them. J Orthop.

[9] Judet R, Judet J, Letournele E (1964). Les fractures du cotyle [Fractures of the Acetabulum]. Acta Orthop Belg.

[10] Pipkin G (1957). Treatment of grade IV fracture-dislocation of the hip. J Bone Joint Surg Am.

[11] Saterbak AM, Marsh JL, Nepola JV, Brandser EA, Turbett T (2000). Clinical failure after posterior wall acetabular fractures: the influence of initial fracture patterns. J Orthop Trauma.

[12] Giannoudis PV, Grotz MR, Papakostidis C, Dinopoulos H (2005). Operative treatment of displaced fractures of the acetabulum. A meta-analysis. J Bone Joint Surg Br.

[13] Tannast M, Najibi S, Matta JM (2012). Two to survivorship of the hip with operatively treated acetabular fractures. J Bone Joint Surg Am.

[14] Meena UK, Tripathy SK, Sen RK, Aggarwal S, Behera P (2013). Predictors of postoperative outcome for acetabular fractures. Orthop Traumatol Surg Res.

[15] Culemann U, Holstein JH, Köhler D, Tzioupis CC, Pizanis A, Tosounidis G, Burkhardt M, Pohlemann T (2010). Different stabilisation techniques for typical acetabular fractures in the elderly–a biomechanical assessment. Injury.

[16] Mears DC, Velyvis JH (2002). Acute total hip arthroplasty for selected displaced acetabular fractures: two to twelve-year results. J Bone Joint Surg Am.

[17] Makridis KG, Obakponovwe O, Bobak P, Giannoudis PV (2014). Total hip arthroplasty after acetabular fracture: incidence of complications, reoperation rates and functional outcomes: evidence today. J Arthroplast.

[18] Suzuki T, Morgan SJ, Smith WR, Stahel PF, Gillani SA, Hak DJ (2010). Postoperative surgical site infection following acetabular fracture fixation. Injury.

[19] Sermon A, Broos P, Vanderschot P (2008). Total hip replacement for acetabular fractures. Results in 121 patients operated between 1983 and 2003. Injury.

